# What do we not know (yet) about recovery colleges? A study protocol on their (cost-)effectiveness, mechanisms of action, fidelity and positioning

**DOI:** 10.1186/s12888-023-05293-8

**Published:** 2023-11-08

**Authors:** Marloes M. C. van Wezel, Christien Muusse, Dike van de Mheen, Ben Wijnen, Wouter den Hollander, Hans Kroon

**Affiliations:** 1 Tranzo Scientific Center for Care and Wellbeing, School of Social and Behavioural Sciences, Tilburg University, Tilburg, the Netherlands; 2 Department of Reintegration and Community Care, Trimbos Institute, Utrecht, the Netherlands; 3 Department of Epidemiology, Data, Evaluation & Monitoring, Trimbos Institute, Utrecht, the Netherlands; 4 Center of Economic Evaluation & Machine Learning, Trimbos Institute, Utrecht, the Netherlands

**Keywords:** Recovery Colleges, Co-creation, Empowerment, Effectiveness, Cost-effectiveness, Fidelity, Mental health care, Protocol, Recovery

## Abstract

**Background:**

Recovery Colleges (RCs) have spread across the globe as a new way of supporting people with mental vulnerabilities in their recovery journey. RCs focus on ‘learning’ rather than ‘curing’ and in that line facilitate a transition from being a passive, dependent patient/client to an active, empowered student learning to live life, despite vulnerabilities. Peer support and co-creation are central in RCs, as peers learn from each other by sharing personal experiences with mental vulnerabilities in an accessible, inspiring and stimulating atmosphere. The implementation of RCs is highly encouraged internationally, and as a result RCs and related self-help initiatives increasingly emerge. However, high-quality research on RCs is scarce and there is a call for thorough investigation of (cost-)effectiveness, mechanisms of action, cross-border fidelity and positioning of RCs. In response, this research project aims to fill these gaps.

**Methods:**

This research project entails (1) a prospective quasi-experimental effectiveness study and economic evaluation, (2) a multifaceted qualitative study to elaborate on the mechanisms of action of RCs for those involved (3) a study to develop a (Dutch) Fidelity Measure of Recovery Colleges, and (4) an organisational case study to describe the positioning of RCs in relation to other mental health care services and community-based initiatives. Following the ideals of co-creation and empowerment in RCs we conduct this research project in co-creation with RC students from Enik Recovery College in Utrecht, the Netherlands.

**Discussion:**

This research project will lead to one of the first longitudinal controlled quantitative evaluations of both cost-effectiveness and effectiveness of RC attendance in a broad sense (beyond attending courses alone). Moreover, we will gather data on a micro level (i.e., impact on RC students), meso level (i.e., organisational fidelity) and macro level (i.e., positioning in the care and support domain), capturing all important perspectives when scrutinizing the impact of complex systems. Finally, we will demonstrate the validity and value of embracing experiential knowledge in science as a complementary source of information, leading to a more profound understanding of what is researched.

**Trial registration:**

The prospective quasi-experimental study has been pre-registered at clinicaltrails.gov (#NCT05620212).

## Background

In the ‘90s, recovery in the context of mental health care has been re-defined as the process of learning how to live a meaningful life despite one’s vulnerabilities [1, 2], moving beyond the narrow-minded definition focusing on reducing clinical symptoms alone [3, 4]. As such, recovery is an active and rich process of gaining and maintaining hope, understanding one’s (dis)abilities, personal autonomy, social identity, meaning and purpose, and getting a positive sense of self. At this time, many mental health care services internationally embrace this definition of recovery as they aim to transform patients from passive, dependent consumers into active producers of care [5].

### Recovery colleges as illumination of changing mental health care

Recently, Recovery Colleges (RCs) emerged as a new way of supporting people with mental vulnerabilities in their recovery process. The first Recovery Education Centers opened in the United States in the ‘90s, after which this innovative approach spread across the globe, with especially strong roots of RCs in the United Kingdom (i.e., UK; [6, 7]). RCs move away from the traditional medical model and adopt an educational model instead, with a focus on ‘learning’ rather than ‘curing’ [8]. In this line, people with mental vulnerabilities are considered active, empowered students in RCs rather than clients^1^. Peer support, co-creation and education are key components of RCs, facilitating an environment where one can learn to deal with their vulnerabilities through sharing experiences and in this way build their own lives from their own strengths [8, 9].

Central to an RC is its curriculum: people can participate in recovery-oriented courses and workshops. Some of these courses are based on existing curricula (e.g., Wellness Recovery Action Planning [WRAP], [10]; Honest, Open, Proud [HOP], [11]; Recovery Is Up to You, [12]), other courses result from co-creation among RC students (e.g., courses about stigma, finding meaning in life). Often RCs facilitate additional opportunities, for example to meet peers in a social meeting place, to work as a volunteer (e.g., in the social meeting place or as co-facilitator of courses) or even to attend a retreat (i.e., a multiple-days overnight stay with a recovery-oriented program). In turn, individuals can be involved in an RC as visitors (i.e., visiting the social meeting place), RC course students (i.e., attending a workshop or training), volunteers, *and* employees. Note that we write ‘and’ instead of ‘or’: individuals can be (and often are) active in multiple ways 2. Although people with mental vulnerabilities are the main target group of RCs, anyone who feels addressed may partake [13].

RCs are deemed promising as attendance has been related to increased well-being, quality of life, achievement of personal recovery goals, and reduced service use and costs [13–15]. In turn, their implementation is highly encouraged (inter)nationally (e.g., [16, 17]). Despite this, an extensive body of high-quality studies investigating RCs is lacking (see e.g., [13, 14]). In response, we here describe the protocol for a multifaceted research project aiming to fill this gap. In the following section we describe our four aims: (1) to investigate the (cost-)effectiveness of RCs, (2) to scrutinize the mechanisms of action of RCs for those involved, (3) to develop a fidelity measure for RCs, and (4) to describe opportunities and risks of RCs as part of the care and support domain.

### Aim one: the (cost-)effectiveness of RCs

High-quality quantitative research on the (cost-)effectiveness of RC attendance is extremely scarce. Although several reviews have been conducted [13–15, 18], the majority of included research did not report empirical data, or were not peer-reviewed [13]. Additionally, empirical studies generally had an uncontrolled (pre-post) design, low sample sizes, and/or unvalidated outcome measures. While there are several partial economic-evaluations investigating costs [19–21], there is a paucity of full economic evaluations of RCs (i.e., investigating costs and effectiveness at once). Hence, there have been repeated recommendations for longitudinal and ideally case-control studies with a higher level of evidence [13, 14].

Empowerment is a surprisingly underassessed outcome measure, while empowering RC students is one of the key principles of the RC model [6, 18]. Notably, initial evidence for increased empowerment after involvement in an RC [22], an RC course [10–12], or other peer-led interventions [23] is promising. To that end, the first aim of this research project is to evaluate the (cost-)effectiveness of RCs, with specific focus on empowerment.

### Aim two: mechanisms of action of RCs

While the body of international research on RCs is still in its infancy, qualitative work is more prevalent than quantitative evaluations [13]. Extant qualitative work provides valuable insights regarding the meaning of RCs (e.g., [24, 25]) and underlying mechanisms of action (e.g., [18, 26]). Combining various sources of information in the form of triangulation is especially fruitful to gain a profound understanding of complex phenomena [27]. To do justice to the complexity of understanding individual recovery processes in a dynamic, interpersonal learning environment as an RC, we therefore believe that it is valuable to conduct research embracing information and observations from multiple perspectives and sources. To that end, the second aim of this research project is to describe what RC attendance means for someone’s recovery journey, and which mechanisms of action give shape to that journey, by means of a multifaceted qualitative study.

### Aim three: the fidelity of RCs

Several authors from the UK have described key principles of the RC model [6, 18, 28, 29], which have been aggregated into the UK RECOLLECT Fidelity Measure by Toney, Knight [8]. However, significant variation exists in the way RCs are given shape, within [30] and outside the UK [31]. To illustrate, in the Netherlands one important deviation from the UK principles regards co-creation. In the UK, RCs are co-created with mental health care professionals [8], while most RCs in the Netherlands are 100% peer-run (Dutch Association of Self-Direction and Recovery [32]). Scrutinizing this different situation in the Netherlands will illuminate possible shared values in RCs across international borders, which strengthens the transformational power of the RC model (see also [33]). In turn, the third aim of this research project is to develop a fidelity measure that highlights core elements of Dutch RCs specifically.

### Aim four: the positioning of RCs

As stated, it is increasingly recognized that adequate health care and support of people with mental vulnerabilities entails more than medically oriented care alone. Importantly, in traditional mental health care services existential and social problems such as meaninglessness and loneliness remain unaddressed, while related existential and social components (i.e., finding back meaning in life and engage in meaningful social relationships) are central to recovery (see for example Johnson [34]). RCs are proposed to be ‘the backbone of the community’, as they fulfil that need for existential and social oriented facilities [17]. Yet achieving a successful collaboration within an adequate integrated care system is complex given the variety and fragmentation of relevant parties involved, not only within the health care domain (e.g., [35]) but also in social services (e.g., [36, 37]). To that end, the final aim of this research project is to scrutinize the collaboration between stakeholders and RCs in the care and support domain.

### Co-research as methodology

To increase the quality of our research, and to operate in line with the philosophy of RCs (i.e., embracing co-creation and empowerment as central elements), this research project is conducted in close collaboration between academic researchers and experiential researchers (i.e., the POP group^3^). The POP group consists of nine experiential researchers at the time of writing, who are visitors, students, volunteers and employees of Enik Recovery College (hereafter ‘Enik’ in short). Enik opened its doors as one of the first Dutch RCs in 2015 in Utrecht, the fourth largest city of the Netherlands, after which numerous similar initiatives followed. Given that Enik is a well-established RC in the Netherlands serving as an example to many others, it is the base site of this research project.

The experiential researchers are involved in every step of the way, from recruitment of participants, study design, data collection and analysis, to writing up and disseminating the results. Including experiential researchers in a research team poses many benefits (e.g., [38–40]): (1) recruiting a more inclusive sample, including participants who may be more difficult to reach otherwise, (2) designing studies to match the participants’ skills and vocabulary (3) designing studies to investigate relevant themes that stem from practice (instead of only from literature and theory), and (4) gaining a more coherent and contextualized understanding of the findings.

## Methods

This research project entails four studies: (1) a prospective effectiveness study including an economic evaluation, (2) a qualitative study, (3) a fidelity study and (4) a positioning study. In this section, the methodology of the four studies is described in accordance with applicable guidelines (effectiveness study, STROBE guidelines, [41]; economic evaluation, CHEERS guidelines, [42]; qualitative studies, SRQR guidelines, [43] and COREQ criteria, [44]).

### Study 1: prospective effectiveness and economic evaluation

The first study focusses on the effectiveness of RCs in terms of recovery-related outcomes (study 1A), and entails an economic evaluation of the cost-effectiveness of RCs (study 1B).

#### Research questions

To evaluate the (cost-)effectiveness of RCs, we pose the following research questions (RQs):

RQ 1: Does attending an RC impact empowerment on the long term, and if so, to what extent?

RQ 2: Does attending an RC impact other recovery related outcomes, such as quality of life, (mental) health, loneliness, satisfaction with treatment and support, and self-stigma on the long term, and if so, to what extent?

RQ 3: What is the cost-effectiveness of RCs?

#### Design

**Fig. 1:**
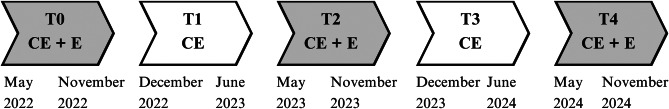
Participant timeline of the five wave study design

Random allocation to conditions is not feasible, since that would counteract the open-to-all accessibility of RCs. To allow for drawing reliable and valid conclusions, we will adopt a quasi-experimental design where RC students (the experimental group) are compared with members of the Dutch panel of people with severe mental illness (i.e., SMI) (the control group), based on propensity score matching. The Dutch panel of people with SMI (hereafter referred to as ‘the panel’) is a national panel with over 1,500 members who have lived experience with mental vulnerabilities. The target group of this panel is specified as “people (aged ≥ 18) experiencing various SMI” and is expected to be similar to the target group of RCs (for more detailed information on the panel, see de Lange, Michon [45]). RC students receive the same questionnaires as members of the panel (i.e., the control group). All participants (in both the experimental and control group) will fill out a survey every six months, for a two-year period (5 assessments in total). To minimize recall bias (e.g., when determining resource use; [46, 47]) and to determine cumulative quality-adjusted life years, outcomes relevant for the economic evaluation were included in all five waves. Relevant measures to determine the effectiveness of RCs will only be included in t_0_, t_2_ and t_4_ to keep the survey length manageable (every year; see Fig. 1).

#### Population

All study participants (both experimental and control group) are people with mental vulnerabilities. RC students will be matched with panel members based on relevant key characteristics through propensity score matching [48].

**Inclusion Criteria.** To be included in the experimental group, people need to be current RC students at the moment of recruitment (in any role, so including volunteers and employees). Moreover, participants need to experience SMI or mental vulnerabilities (both self-reported or diagnosed).

**Exclusion Criteria.** Participants are not eligible if they do not sufficiently master the Dutch language to properly understand and fill out the surveys (even with assistance from the POP group experiential researchers). Specifically for the control group, participants will be excluded from the matching procedure if they are involved in an RC or similar initiatives (i.e., recovery-oriented consumer run organisations) at time of the baseline measurement. If panel members visit one of the included RCs at the time of recruitment, they will be allocated to the experimental group.

#### Procedures

**Recruitment.** The recruitment of participants in this study is two-fold. First and most importantly, participants for the experimental group will be recruited from four RCs in the Netherlands. Enik is the base site of this research project, and we additionally include three other RCs for two reasons: (1) to increase the generalizability and quality of our data and (2) to reach a sample size that is sufficiently large to draw conclusions with a satisfactory certainty. The three additional RCs were selected in collaboration with the co-founder of Enik, and were considered to be well-established and based on a similar philosophy.

RC students will be invited via physical (i.e., posters, flyers) and digital promotion material (i.e., email, newsletter, information page on the RC website, video). Furthermore, facilitators of RC workshops will recruit participants in their groups, and the research team (incl. POP group) will actively recruit participants in the RC’s social meeting place and team meetings. In addition, online and physical information sessions will be hosted to provide information about participation, and assistance with enrolling. When willing to participate, participants receive a digital information letter, provide informed consent and fill out an online application form. Members from the POP group can assist participants in this process if necessary.

Second, as part of regular maintenance of the panel, recruitment for the panel is done via physical and digital promotion materials, which will be distributed among the target population in mental health services’ divisions for people with SMI, and in public (e.g., online advertisements or advertisements in newsletters).

**Between-Group Differences.** RC students are considered similar to the panel’s target population in many ways (e.g., SMI, use of care), yet the most important between-group difference is that those in the experimental group are (or have recently been) actively involved with one of the participating RCs, while members of the panel that are included as controls are not.

**Matching.** To control for potential confounding given the natural (non-randomized) assignment of conditions, we will use propensity score matching and/or exact matching. All participants who have completed at least two waves will be considered for the matching procedure.

Variables that will be used for the matching are gender, age, nationality, level of education and diagnoses (psychotic disorders, mood disorders, anxiety disorders, personality disorders, substance use disorders, neurodevelopmental disorders, eating disorders). If someone does not have an official diagnosis, we will use self-reported diagnosis as a proxy. Although potentially relevant, duration of service use will not be used as matching variable since true baseline data are unavailable; the experimental group may be already active at the RC for a significant amount of time at t_0,_ which may have impacted service use. Each participant from the experimental group will be matched to one or more participants from the control group. Given the large sample size of the control group and the limited number of matching variables, we will likely retrieve multiple good matches per RC partaker, reducing uncertainties and increasing reliability.

#### Variables of interest study 1A and 1B

For clarity, the variables of interest are reported here for the effectiveness study (1A) and the economic evaluation (1B) separately. Demographic and descriptive variables are relevant for both studies 1A and 1B.

**Demographic and Descriptive Variables.** Basic demographics (gender, age, nationality, level of education, employment status, housing), and SMI-specific variables (mental health problems; self-reported and officially diagnosed, duration of mental health problems, health care use) will be used to describe the sample. As noted, many of these variables will be used to match participants from the control group (panel members) with participants from the experimental group (RC students). Furthermore, participants in the experimental group will indicate in what way and how frequently they are active at the RC.

#### Variables of interest for study 1A: effectiveness

**Primary Outcome Measure: Empowerment.** The main dependent variable, empowerment, will be operationalized by the Netherlands Empowerment List (NEL; [49]). Originally, the NEL consists of six subscales (social support, professional help, connectedness, confidence and purpose, self-management and caring community). To keep the survey length manageable, we have selected three subscales as an operationalization of personal empowerment, following Boevink, Kroon [49]: confidence and purpose, connectedness and self-management. This selection “describes feelings, competencies and actions reflective of personal empowerment” [49]. The NEL has shown good internal consistency (Cronbach α = 0.94), aspects of validity, reproducibility (Cronbach α = 0.79), and responsiveness [49, 50]^4^. Example items are “I think of myself as a person worth something” (confidence and purpose), “I regularly meet people outside my home” (connectedness), and “I am able to set my boundaries” (self-management). Items are answered on a 5-point Likert scale (‘1’ = Strongly disagree, ‘5’ = Strongly agree).

**Secondary Outcome Measures.** This study investigates five secondary outcomes.

***Quality of Life.*** To measure quality of life the 8-item Maastricht QoL Scale [51] will be used. Participants rate their satisfaction about their living situation, social contacts, daily activities, financial situation, mental health, physical health, received care, and their life as a whole. An example item is “How satisfied are you with your daily activities?”. Items are answered on a 7-point Likert scale (‘1’ = Not at all satisfied, ‘7’ = Extremely satisfied).

***Mental Health.*** To measure mental health, the MHI-5 [52] will be used. Five items inquire about the frequency of experiencing negative and positive affect, anxiety and depression during the past four weeks. An example item is “How much of the time, during the last four weeks, have you felt calm and peaceful?”. Items are answered on a 6-point Likert scale (‘1’ = Constantly, ‘6’ = Never).

***Loneliness***. Loneliness can be defined as a state of mind where one is unsatisfied with the quantity and quality of their social network [53]. The 11-item DeJong Gierveld Loneliness Scale [54] will be used to operationalize loneliness. This scale is comprised of two subscales measuring emotional loneliness (6 items) and social loneliness (5 items; [55]). Example items are “I miss having a really close friend” (emotional loneliness), and “I miss having people around me” (social loneliness). Items are answered on a 5-point scale (‘1’ = Yes!, ‘2’ = Yes, ‘3’ = More or less, ‘4’ = No, ‘5’ = No!).

***Satisfaction with Care and Support.*** To measure how satisfied participants are with received care and support, we will use an adapted inventory from Menting [56]. Originally this inventory is targeted at people with chronic illness, so we adapted the items to concern mental health care specifically. Participants provide a rating for specific health care providers (e.g., general practitioner, psychiatrist) ranging from 1 to 10, with a higher score representing a higher satisfaction with received care and support. Participants only provide a rating for specific health care providers of whom they have received care in the past 12 months. In addition to these ratings from the adapted inventory [56], participants also rate their general satisfaction with received care and support from care providers and their social network. These two items are answered on a 5-point Likert scale (‘1’ = Very unsatisfied, ‘5’ = Very satisfied). Finally, we added two items inquiring the extent to which the received care and support from care providers and their social network matches participants’ needs, on a scale from 1 to 10 (‘1’ = Extremely bad, ‘10’ = Excellent).

***Self-Stigma***. Self-stigma, or internalized stigma, refers to the application of negative stereotypes and discrimination to oneself, and will be operationalized by the 10-item Internalized Stigma of Mental Illness scale (ISMI-10; [57]). An example item is “Mentally ill people tend to be violent”. Items are answered on a 4-point Likert scale (‘1’ = Strongly disagree, ‘4’ = Strongly agree).

#### Variables of interest study 1B: economic evaluation

***Resource Use.*** To assess (informal) care use of participants, the Trimbos and iMTA questionnaire on Costs associated with Psychiatric illness (TiC-P; [58]) will be used, with a recall period of three months. This includes questions on medication and contact-hours with care providers but also questions about informal care received from one’s own social network (e.g., family, acquaintances, neighbours). To assess community outcomes such as employment, again the TiC-P [58] will be used. This includes questions on whether participants have employment or are active as volunteer, absenteeism and presenteeism of their (un)paid jobs. The recall period for absenteeism and presenteeism is four weeks. Housing status (e.g., living independently, living in a hospital/sheltered or supported housing residence, having no permanent residence) will also be determined by one item that we formulated (not in the TiC-P).

***Quality-Adjusted Life Years.*** In line with the Dutch guidelines for economic evaluations, quality-adjusted life years (i.e., QALYs) will be computed using utilities derived from the EuroQol-5-dimensions-5-level-questionaire (EQ-5D-5 L; [59]). The EQ-5D-5 L consists of six items, of which five inquire about specific restrictions in daily life (i.e., health states), with regards to mobility, self-care, usual activities, pain/discomfort and anxiety/depression. Possible answers on these items range from 1 to 5, where ‘1’ = Indicating no problem and ‘5’ = Indicating unable to/extreme problems. One final item regards the general health state (the EQ VAS: “We would like to know how good or bad your health is TODAY. Mark an X on the scale to indicate how your health is TODAY”). This general health state is represented by a number between 0 and 100, where ‘0’ = The worst health you can imagine, and ‘100’ = The best health you can imagine. The health states from the EQ-5D-5 L will be computed into utilities using the Dutch tariffs of EuroQol [60]. By means of the area under the curve method, time periods between assessment waves will be weighted by these utilities, which results in QALYs adjusted over the timeframe of 24 months. An overview of all measures can be found in Table 1.

**Table 1:** Overview of all variables of interest and its matching instruments as used in Study 1

Measurement level	Variable of interest	Instrument	Reference
Primary	Empowerment	NEL	Boevink, Kroon (49)
Secondary	Quality of life	Maastricht QoL	Drukker, Bak (51)
	Mental health	MHI-5 (from RAND-36)	Berwick, Murphy (52)
	Loneliness	DeJong Gierveld Loneliness Scale	de Jong-Gierveld and Van Tilburg (54)
	Satisfaction with care and support	Adapted inventory	Menting (56)
	Self-stigma	ISMI-10	Boyd, Otilingam and DeForge (57)
Economic evaluation	Resource use	TiC-P	Hakkaart-Van Roijen (58)
	Quality-adjusted life years	EQ-5D-5 L	Rabin and Charro (59)

*Note*. All measures are self-reported

#### Sample size

The required sample size to reach at least 80% power is determined through simulation using the R packages simstudy [61] and faux [62]. To our knowledge, no previous studies included empowerment as primary outcome variable, so our estimation of the expected effect size of Cohen’s D is based on previous work on peer support effectiveness [63] and on RC effectiveness [20]. These effect sizes were small. In our case, the hybrid nature of RC attendance and the selection of NEL subscales may potentially lower the capability to detect clinically relevant changes^5^. On the other hand, the NEL has proven to be sensitive to change in recovery-oriented practices (e.g., medium effect size in Tjaden, Mulder [64] after 18 months). All this considered, we assume an effect size of Cohen’s D = 0.3. Assuming Cohen’s D = 0.3, a Pearson’s correlation coefficient of 0.6 between subsequent measurements [64] and 30% dropout at t_4_ we expect to detect a significant difference in 82.8% of the time between the control and experimental group when a total 250 participants are to be included. Participants for the control group will be recruited from the existing panel through propensity score matching, hence the study is appropriately powered when 125 participants for the experimental group have been included and matched to 125 control participants.

#### Analyses

In our primary (cost-)effectiveness analyses we will adopt an intention-to-treat approach. Since RCs attendance is voluntary, RCs are available-to-all and are increasingly implemented in the Netherlands, we are aware that cross-overs during the study’s timeframe are likely to occur (e.g., individuals who were active RC students at t_0_ but not anymore at t_3_, or vice versa). However, given the pragmatic nature of the study, we will not take cross-overs into account and treat participants according to their original condition. Depending on the number of actual cross-overs we might explore their impact in a sensitivity analysis. Another sensitivity analysis will concern the nature of RC attendance. Given the hybrid nature of RC attendance in the Netherlands (e.g., one can follow courses, visit the social meeting ground, volunteer) we will compare effects of RC attendance in the broad sense with effects of RC attendance for students who participate in courses (more in line with international literature). Potential baseline between-group differences that were not accounted for in the matching procedure will be checked and controlled for if needed. We will adopt single imputation nested in bootstrapping to deal with missing values and skewed data [65]. All analyses will be conducted in SPSS version 29 + and/or R version 4.2+. In the following sections, the specific analyses to examine the effectiveness and economic evaluation of RCs are described separately.

#### Analysis study 1A: effectiveness

To determine the effectiveness of RC attendance we will conduct multi-level regression analyses with three levels: (1) repeated observations (2) within participants and (3) participants in pairs of intervention and propensity score matched controls. While participants in the experimental group can be considered to occupy multiple sites (i.e. a multisite study), participants from the control group strictly seen cannot. Hence, through likelihood ratio testing it will be determined whether including an additional fourth level (random intercept) for site leads to a significant better model fit, in which all participants in the control group will be considered to reside at a single unique site. As no true pre-intervention randomization is possible, the effect analyses will be performed slightly modified to as what is recommended by Twisk, Bosman [66] in that the fixed effect for group will be included in the repeated measures models, alongside the fixed effect for time and the interaction between those. In addition, the models will be fitted without intercept, aiding in interpretation of potential (not intervention related) differences between the control and experimental group. All multi-level analyses will be performed using the R packages lme4 [67] and lmerTest [68].

#### Analysis study 1B: economic evaluation

The economic evaluation will constitute both a cost-effectiveness analysis (CEA) and a cost-utility analysis (CUA). In line with the Dutch guidelines, a societal perspective will be adopted indicating that all costs should be included, regardless of who carries these costs [69]. Hence, four types of costs will be included (all expressed in Euros), namely (1) intervention costs, (2) patient and family out of pocket costs, (3) mental health care services costs and (4) costs due to productivity loss. Intervention costs will be estimated with input from experts coordinating RCs. Patient and family out of pocket costs refer to costs from help and support provided by the informal network of someone [70]. To determine costs the most recent costing manual will be used (at the time of writing this is [70]), with applied indexation if needed. Finally, costs of productivity loss include costs of absenteeism and presenteeism (i.e., being present at work but less efficient) of paid employment and volunteer jobs and will be determined by means of the friction cost method [71]. Since our data collection covers a time period of two years, costs will be indexed to the starting year 2022.

For the CEA, the primary outcome will be a proportion of responders based on the NEL after 24 months, which refers to whether or not participants (both experimental and control group) show a significant difference in self-reported empowerment at t_4_ as compared to t_0_. This variable will be dichotomized using the reliable change index by Jacobson and Truax [72]. For the CUA, the primary outcome will be the total incremental QALYs during the study’s timeframe of 24 months. Cumulative costs will be calculated based on this same time period.

To determine the cost-effectiveness of RC attendance, incremental cost-effectiveness ratios (ICERs) will be calculated for both the CEA and CUA, following Drummond, Sculpher [73]: ICER = (Costs^RC^ – Costs^control^)/(Effects^RC^ – Effects^control^). These ICERS are representative of average incremental costs associated with one additional unit of the measure of effect. Thus, the ICERs of the CEA represent the incremental costs per significant change in self-reported empowerment, and the ICERs of the CUA refer to incremental costs per QALY gained. Analysis will be done according to the intention-to-treat principle. We will use single imputations nested in non-parametric bootstrapping (N > 2500 bootstrapped samples) combined with seemingly unrelated regression equations [65]. Next, incremental costs, incremental effects and ICERS will be calculated. Sensitivity analyses will be conducted to assess the robustness of the findings, for example by running an analysis from the health care perspective (excl. patient and family costs, informal costs and productivity losses) [73].

### Study 2: multifaceted qualitative study

While the first study provides insights in the (cost-)effectiveness of RCs, we also want to know more about the deeper meaning of an RC for those involved. To this end, a multifaceted qualitative study will be conducted, entailing a triangulation of observational data, twin-interview data and diary data.

#### Research questions

In this in-depth qualitative study we aim to answer the following research questions:

RQ 4: What is the meaning and significance of an RC for those involved?

RQ 5: What are the mechanisms of action (both promoting and impeding factors) of an RC that give shape to the (personal) recovery process of those involved?

RQ 6: How are RC values translated into practice?

#### Population

The target population will be similar to the population of Study 1 (experimental group), with two adjustments. First, the recruitment for this qualitative study will be limited to Enik only, to allow for a thorough in-depth investigation. Since the four selected RCs in Study 1 are considered comparable, we believe this choice is to justify. Second, both active and ex-RC students will be invited to participate.

#### Procedures

**Study 2A: Participatory Observations.** The first author will engage in participatory observations in various occasions at Enik, including team meetings, courses, volunteering and the social meeting place. The goal of these observations is fourfold; (1) getting acquainted with and remain informed about the organisation, both at the daily operational level and the managerial level, (2) building rapport with RC students, (3) experiencing what it is like to partake in an RC in various ways, and (4) collecting data to answer the posed research questions. Participatory observations provide highly valuable information that is not structured by researchers, within the natural context of RCs, in the direct moment of occurrence (unlike for example interviews where participants are asked to reminisce their experiences outside the original context, prompted by interviewer’s questions; [74]). The participatory observations will lead to various data, such as field notes, internal documents and informal interview reports. The researcher will always be transparent about the research aims and gathers informed consent when possible.

**Study 2B: Twin-Interviews.** The interviews will be conducted in duos (hence “twin-interviews”): one academic researcher interviews together with one experiential researcher from the POP group. The experiential researchers will receive training on interview techniques. Involving experiential researchers as co-interviewers aims to improve the quality of the interview data, given that they can pose important questions from their own experiential knowledge of RC attendance – something that academic researchers do not necessarily have.

***Recruitment.*** We aim to interview twenty-five participants who form a rich palette of RC students: course students, visitors of the social meeting place, participants of a retreat, volunteers, employees and ex-students. To ensure an adequate representation of all these roles, recruitment will be small-scale and based on personal invitations from the (co-)researchers and RC staff (i.e., purposive sampling and snowballing). Participants provide written informed consent before participating.

***Materials.*** The twin-interviews will focus on the way students experience the core values of RCs (such as connectedness, empowerment, equality, reciprocity and free space^6^), and how these experiences give shape to their recovery journey. The semi-structured interviews stimulate to share both positive and negative experiences, both gains and struggles. The interview protocol will be developed in collaboration with the POP group. All interviews will be audio-recorded and transcribed verbatim. Pseudonymized transcripts will be stored on a highly-secured drive and shared with the participants for a member check before being analysed.

**Study 2C: Diary Study.** The diary study will be a follow-up on the twin-interviews and aims to get more insights in the role of an RC in the daily lives of participants. While interviews mostly regard reflections of past time frames and key events (e.g., the period one was an active RC partaker), diary data is administered on the spot, which may counter retrospective recall problems [76]. Furthermore, diaries allow to investigate the impact of RC attendance in- and outside the RC context, which is important since the effectiveness of interventions is also determined by factors beyond the interventions (e.g., having a social network or involvement with mental health care services; [77]).

The philosophy of empowerment will be embraced in the study’s design, as participants can design their own personal diary. Out of pre-defined themes participants can choose how many and which themes they would like to monitor. The diary study procedure will be threefold: (1) intake interview to select the desired themes, (2) diary period of 1 month (possibility to expand with another month), (3) evaluation interview.

After the intake the researcher sets-up the personal diary and the one-month study period begins. Diaries will be programmed in Qualtrics and distributed via e-mail. Participants receive a diary every other day (so 3–4 times per week) and have 24 h to fill it out, at a time that suits them best. After the study period of one month, participants can decide to fill out diaries for an additional month. When they decide to stop their diary period, an evaluation interview will be scheduled, in which the participant and (co-)researchers search for patterns or important observations stemming from the diary entries. Participants will bring their own diary data to the interview by means of a personalized RShiny dashboard, to enhance feelings of data ownership. These evaluation interviews will again be in twin-interview set-up and follow the same data processing procedures as study 2B (audio-recorded, transcribed verbatim, pseudonymized, member checked).

***Recruitment.*** Participants from the twin-interviews will be invited to participate in the diary study when the interview is concluded. Since this study is considered an elaboration of the interviews, there is no strived sample size. Participants provide written informed consent prior to the intake.

***Materials.*** In collaboration with the POP group, pre-defined themes will be developed. These themes are operationalized by one bipolar question (e.g., “Did you feel supported in the past 24 h?” Predominantly yes/Predominantly no) and an open text field to elaborate (e.g., by providing examples, describing meaningful events and feelings and thoughts accompanying those).

#### Analysis

The data analysis of this qualitative study (Study 2A, 2B and 2C) will be a collaborative [78], ongoing iterative process, where data collection and data analysis are intertwined. The academic and experiential researchers will move back and forth between empirical data, theory and new data collections, following the grounded theory approach [79, 80]. Interpretive and inductive coding will be supplemented with focused coding and theoretical coding when necessary. The majority of the analysis will be conducted using MAXQDA software yet analogue coding sessions with the POP group will be organized too.

#### Study 3: fidelity of Dutch RCs

The third study of this research project entails adapting the UK Fidelity Measure [8] to the Dutch context (phase 1) and testing that adapted Dutch Fidelity Measure for Recovery Colleges (phase 2). When we mention the Fidelity Measure in the continuation of this text, we refer to this adapted Dutch version.

#### Research questions

In this fidelity study we aim to answer the following research questions:

RQ 7: What are the core elements of RCs in the Netherlands?

RQ 8: Is the model of Dutch RCs different from RCs in the UK? And if so, in what way?

RQ 9: What is the fidelity of the different RCs in the Netherlands?

#### Population

This study will invite managers and peer trainers from numerous initiatives to facilitate an open discussion about what constitutes an RC in the diverse Dutch landscape of recovery-oriented practices. Moreover, along the procedure of this study a team of experts will be invited to think along (e.g., from the Dutch mental health care interest group Mind, the Dutch Association for Self-direction and Recovery and methodological experts).

**Inclusion Criteria.** In this study two different types of inclusion will be relevant: being eligible for the focus groups in phase 1 and being eligible for the testing in phase 2. In phase 1, proposed inclusion criteria for an initiative to be considered an RC are at least: (1) offering recovery-oriented self-help courses, (2) being accessible and available to all, and (3) being organized based on valuing experiential knowledge. However, the final inclusion criteria will be determined after consulting the team of experts. In phase 2 similar inclusion criteria will be used, though with a wider scope to invite organisations with indefinite or unclear eligibility too. Namely, a wider scope will provide more information on the suitability of the Fidelity Measure for recovery-oriented practices.

#### Procedures

In phase 1 the UK Fidelity Measure [8] will be translated to Dutch and discussed in focus groups in the light of Dutch RCs specifically. Within these focus groups core elements of Dutch RCs will become apparent, though there is also room for variation (similar to the Type I and Type II in the UK Fidelity Measure). Experiential researchers from the POP group will be trained to co-facilitate these focus groups together with the academic researchers. Focus groups will be organized online via Microsoft Teams to facilitate national participation, audio-recorded, transcribed verbatim and pseudomized. The focus group data will be incorporated into a final version of the Fidelity Measure in close collaboration with the POP group and the team of experts.

In phase 2 the Fidelity Measure will be tested in as many recovery-oriented practices in the Netherlands as possible. Similar to the UK Fidelity Measure, this measure will be a self-report tool and is to be filled-out by the RC manager only. Following Toney, Knight [8], participants can provide written feedback on face and content validity, comprehensiveness, acceptability and usability after filling out the measure. Depending on the number of recovery-oriented practices in the Netherlands that we can identify and are willing to participate, a subsample may be randomly selected to engage in a thinking-out-loud procedure, where the participant shares their thoughts when filling-out the measure. The researchers may also conduct several site visits to better understand what different recovery-oriented practices look like.

**Recruitment.** To obtain an overview of relevant initiatives in the Netherlands we will use multiple sources, such as overviews from the collaborating interest groups in our expert team. In addition we will use our own network, LinkedIn and Google Search to locate eligible initiatives. Participants for the focus groups in phase 1 will then be invited via email directed to the RC managers, who are invited to recruit peer trainers too, for a selection of the initiatives found. An information letter will be attached to the email for this snowballing sampling method. Invited RCs for phase 1 will be heterogenous in terms of longevity (new vs. well-established), recovery-focus (narrow vs. wide), organisational (in)dependency (integrated in mental health care services vs. independent), and geographical density (high vs. low dense areas). Recruiting RC managers to test the Fidelity Measure in phase 2 will be via email in a similar way. All participants provide written informed consent before participation.

#### Analysis

Like the data collection, the data analysis consists of two phases. In phase 1 the focus group data will be thematically analysed to identify which substantial amendments should be made to reflect the Dutch context. The pre-existing items from the UK Fidelity Measure will be used as a basis, but additional items may be included if necessary. Both the POP group and the team of experts will be included when identifying and implementing the needed amendments.

In phase 2 the extent to which the Fidelity Measure suits the Dutch context will be evaluated through thematic analysis of the written feedback and possibly thinking-out-loud data (again in close collaboration with the POP group). The fidelity of Dutch RCs will be determined in a way suitable to the final measure, depending on amendments made. Both thematic analyses will be conducted in MAXQDA and in collaboration with the POP group in a similar way as in Study 2.

### Study 4: RCs in the care and support domain

As a final study of this research project, we will zoom in on the positioning of RCs in the care and support domain.

#### Research questions

RQ 10: What does the collaboration between stakeholders and RCs look like in the care and support domain?

RQ 11: Which opportunities arise from a successful collaboration between stakeholders and RCs in the care and support domain?

RQ 12: Which pitfalls or dilemmas can be identified in the collaboration between stakeholders and RCs in the care and support domain?

#### Population

Given the multidimensional approach to mental health and well-being adopted by RCs, they interface with a range of sectors, within and outside mental health care (i.e., the care and support domain)^7^. To that end, we will interview stakeholders from a variety of fields, including mental health care services, community and social services initiated by welfare and housing organisations, municipality services, and of course the RC itself. This also includes stakeholders relevant for funding, such as health insurers and municipalities.

#### Procedure

To investigate what collaboration between stakeholders and RCs looks like, we will conduct several focus groups with individuals involved in relevant organisations. To gain a profound understanding of possibilities and difficulties we will adopt a case-study design, zooming in on the positioning of Enik. Given that Enik is a well-established initiative and comparable to the other included RCs from Study 1, we believe that this is a valuable case to scrutinize opportunities and barriers in sustainable RC implementation.

**Recruitment.** We will recruit individuals involved in various layers (e.g., executives, case workers, volunteers) of the organisation that are invited to participate in the focus groups. This way we will gather information on a managerial level and on an operational level. Participants will be personally invited via email, social media or phone calls and provided with an information letter (i.e., purposive sampling). Snowballing within organisations may be required to reach individuals from various layers, for which we will invite executives to recruit using our information letter. All participants will provide informed consent prior to participation.

**Materials.** Following our co-research methodology we will develop a topic list to be used in the focus groups in collaboration with the experiential researchers from the POP group. This way we will not only answer questions that are relevant on an organisational level but also questions that are more directly linked to the practice which these organisations affect.

#### Analysis

We will use initial inductive coding after which we thematically analyse the focus group data using MAXQDA. To collaborate with the POP group we will adopt a similar approach as in Study 2 and 3.

## Discussion

This paper describes the study protocol for assessing the (cost-)effectiveness of RCs, scrutinizing the mechanisms of action of RCs, as well as evaluating the fidelity and positioning of RCs. By conducting this research we contribute to the field in three important ways. First, to our knowledge we will conduct one of the first longitudinal controlled quantitative evaluations of both cost-effectiveness and effectiveness of RC attendance. Notably, we not only include RC students in self-help courses as study participants, but widen the scope to investigate impact of other activities students conduct in an RC, which is rarely done. This is important given that RCs facilitate possibilities to explore fluid roles and opportunities that suit your recovery journey at that particular moment, reaching beyond participating in self-help courses.

While the richness of possibilities for RC students is at the core of the RC concept, it also complicates understanding which aspects of the RC are effective, for who, in what context, using quantitative methodologies alone. To that end, a second contribution to the field is our use of a mixed-methods design, which will revenue profound data on the meaning and mechanisms of action of RCs. The obtained qualitative data will aid the interpretation of our quantitative findings, besides being valuable on their own. Given our various contextual studies, we will gather data on a micro level (i.e., impact on RC students), meso level (i.e., organisational fidelity) and macro level (i.e., positioning in the care and support domain), capturing all important perspectives when scrutinizing the impact of complex systems [81].

Third, we will demonstrate the validity and value of integrating experiential knowledge in science as we closely collaborate with RC students (i.e. the “POP group”) in all stages of this research project. Embracing experiential knowledge as a valid source of information leads to a more profound understanding of research findings through ongoing reflections from multiple perspectives, increasing scientific rigor. It also reduces the gap between research and practice. As a result, empowering peers as valued experiential researchers leads to research that is relevant for both science and practice.

Of course, this research project will likely encounter several challenges. First of all, while RCs aim to facilitate an inclusive environment where individuals with all degrees of vulnerabilities can work on their recovery, it is the question to what extent such variety of individuals are actually reached by the RCs. For example, care providers may hold false beliefs that recovery is less relevant for ‘their patients’ [1], or may be concerned about too high expectations in a ‘college’ [82]. Moreover, regardless of whether the actual RC population is representative of its intended target population, the question remains whether our research sample is, too. To illustrate, one common selection bias in mental health research stems from non-participation of people with relatively more severe difficulties (e.g., [83]). Transferability of our findings to the target population of RCs is in that line yet to be determined and should be carefully considered.

Second, keeping participants engaged within a research project can be challenging, especially when adopting a longitudinal survey design with long in-between periods of non-activity (in our case, 6 months). Longitudinal epidemiological research in the field of psychiatry is posed to be especially prone to significant (selective) attrition rates, reducing statistical power and potentially introducing bias when drop-outs are selective [84]. Therefore, repeatedly communicating the importance of participation and having personal contact with participants when possible will have our special attention during the data collection to minimize attrition rates. We also believe that the multi-faceted mixed-methods approach of this research project makes it more attractive for participants to stay involved, as we provide an opportunity to let their voices be heard beyond surveys alone.

To conclude, the implementation of RCs and similar recovery-oriented practices is booming in attempts to redesign mental health care services so that it becomes a sustainable, affordable, effective and person-centred ecosystem. Thorough high-quality research into whether and how RCs can contribute to recovery and how RCs can be successfully implemented is therefore of great importance.

## Data Availability

Not applicable.

## Footnotes

1 Please note that whenever we refer to ‘students’ in this article, we refer to people who attend courses and workshops at RCs, rather than students of colleges or universities.

2 In the remainder of this manuscript, individuals involved in RCs in any way are referred to as RC students, which is the most common term in international RC literature. Note that RC students may also include staff since they are peers too.

3 POP stands for *Peer Onderzoeker Perspectief*, which translates to ‘Peer Researcher Perspective’ in English.

4 These reported psychometric properties regard the NEL as a whole, yet we expect that the psychometric properties for the chosen shortened version of three selected subscales will be similar and therefore acceptable.

5 We reanalyzed outcomes on the dataset from Tjaden, Mulder [64], with the three selected subscales of the NEL as primary outcome instead of the whole instrument (as reported in the paper). This lead to a modest drop of the effect size from d = 0.53 (total scale) to d = 0.43 (three subscales).

6 Free space refers to both the inner space nested in every individual and the explicit space facilitated by RCs in which people (re)discover vitality, meaning, and empowerment fueled by their unique needs and desires ([75]. Boertien D, Harmsen K. Herstelacademie - vrijplaats voor eigen ontwikkeling. Utrecht: Werkplaats Herstelondersteuning; 2017.)

7 In the Netherlands, a part of the community and social services is strongly related to the mental health care domain, as governmental subsidies (so-called ‘*WMO funding*’) are available to arrange support for people with mental vulnerabilities.

## List of abbreviations

RC: Recovery College
UK: United Kingdom
WRAP: Wellness Recovery Action Planning
HOP: Honest Open Proud
RECOLLECT: Recovery Colleges Characterisation and Testing
POP: Peer Onderzoekers Perspectief *[Peer Researchers Perspective*
STROBE: STrengthening the Reporting of Observational studies in Epidemiology
CHEERS: Consolidated Health Economic Evaluation Reporting Standards
SRQR: Standards for Reporting Qualitative Research
COREQ: COnsolidated criteria for REporting Qualitative studies RQ = Research Question
SMI: Severe Mental Illness
CE: Cost-Effectiveness
E: Effectiveness
NEL: Netherlands Empowerment List
QoL: Quality of Life
MHI: Mental Health Inventory
ISMI: Internalized Stigma of Mental Illness
iMTA: Institute for Medical Technology Assessment
TiC-P: Treatment Inventory of Costs in Patients with psychiatric disorders
QALY: Quality-Adjusted Life Years
EQ-5D-5L: EuroQol-5-Dimensions-5-Level-questionaire
EQ VAS: EuroQol Visual Analogue Scale
CEA: Cost-Effectiveness Analysis
CUA: Cost-Utility Analysis
ICER: Incremental Cost-Effectiveness Ratio
WMO: Wet Maatschappelijke Ondersteuning *[Law of Social Support]*
